# Influence of the Phenological State of in the Antioxidant Potential and Chemical Composition of *Ageratina havanensis*. Effects on the P-Glycoprotein Function

**DOI:** 10.3390/molecules25092134

**Published:** 2020-05-02

**Authors:** Trina H. García, Claudia Quintino da Rocha, Livan Delgado-Roche, Idania Rodeiro, Yaiser Ávila, Ivones Hernández, Cindel Cuellar, Miriam Teresa Paz Lopes, Wagner Vilegas, Giulia Auriemma, Iraida Spengler, Luca Rastrelli

**Affiliations:** 1 Center for Natural Product Research, Faculty of Chemistry, University of Havana, Havana 10400, Cuba; trina@fq.uh.cu; 2 Department of Chemistry, Federal University of Maranhão, São Luis, Maranhão 65080-805, Brazil; claudiarocha3@yahoo.com.br; 3 Department of Pharmacology, Institute of Marine Sciences (ICIMAR), Loma 14, Alturas del Vedado, Plaza de la Revolución, Havana 10400, Cuba; ldelgadoroche@gmail.com (L.D.-R.); idania.rodeiro@infomed.sld.cu (I.R.); ivones.hernandez@informed.sld.cu (I.H.); c.cuellar@gmail.com.br (C.C.); 4 Institute of Ecology and Systematics, Havana 10400, Cuba; y.avila@ecologia.cu; 5 Laboratory of Antitumor Natural Substances, Department of Pharmacology, Institute of Biological Sciences, Federal University of Minas Gerais (UFMG), Belo Horizonte 31207-90, Brazil; mtpl@icb.ufmg.br; 6 Experimental Campus of Sao Vicente, UNESP-Sao Paulo State University, Sao Vicente, Sao Paulo 11.330-900, Brazil; wagner.vilegas@unesp.br; 7 Department of Pharmacy, University of Salerno, Via Giovanni Paolo II, 84084 Fisciano (SA), Italy; gauriemma@unisa.it

**Keywords:** *Ageratina havanensis*, flavonoids, UPLC-ESI-MS/MS, P-glycoprotein, antioxidant potential

## Abstract

*Ageratina havanensis* (Kunth) R. M. King & H. Robinson is a species of flowering shrub in the family Asteraceae, native to the Caribbean and Texas. The aim of this work was to compare the quantitative chemical composition of extracts obtained from *Ageratina havanensis* in its flowering and vegetative stages with the antioxidant potential and to determine the effects on P-glycoprotein (P-gp) function. The quantitative chemical composition of the extracts was determined quantifying their major flavonoids by UPLC-ESI-MS/MS and by PCA analysis. The effects of the extracts on P-gp activity was evaluated by Rhodamine 123 assay; antioxidant properties were determined by DPPH, FRAP and inhibition of lipid peroxidation methods. The obtained results show that major flavonoids were present in higher concentrations in vegetative stage than flowering stage. In particular, the extracts obtained in the flowering season showed a significantly higher ability to sequester free radicals compared to those of the vegetative season, meanwhile, the extracts obtained during the vegetative stage showed a significant inhibitory effect against brain lipid peroxidation and a strong reductive capacity. This study also showed the inhibitory effects of all ethanolic extracts on P-gp function in 4T1 cell line; these effects were unrelated to the phenological stage. This work shows, therefore, the first evidence on: the inhibition of P-gp function, the antioxidant effects and the content of major flavonoids of *Ageratina havanensis*. According to the obtained results, the species *Ageratina havanensis* (Kunth) R. M. King & H. Robinson could be a source of new potential inhibitors of drug efflux mediated by P-gp. A special focus on all these aspects must be taking into account for future studies.

## 1. Introduction

Polyphenols, particularly flavonoids, have been widely studied for their bioactive properties and their strong antioxidant effects are well noted [1,2,3]. In addition, some plant antioxidant polyphenols exhibit anti-neoplastic activity by multiple pathways, including their potential to intensify the action of cytostatic drugs, through the attenuating the multidrug resistance (MDR) phenomenon [4]. The best recognized and the most frequent cause of the MDR involves an increased activity of ATP-binding cassette family transporters (ABC) [5]. A large number of compounds have been identified as MDR suppressors by interfering with the P-gp-mediated export of chemotherapeutic agents [6,7,8,9]. However, in many cases the high toxicity of these substances has limited their use [10]. Thus, natural antioxidants have been identified as novel potential candidates [11,12,13,14,15,16]. 

Different studies show that plants of the genus *Ageratina* contain a variety of flavonoids. Particularly, in our previous research studying the extracts obtained from the leaves of *Ageratina havanensis* (Kunth) R. M. King & H. Robinson, growing in Cuba, by chromatographic, spectroscopic and spectrometric methods, we identified a significant presence of flavonoids and their glucosides [17,18]. We also determined that the qualitative composition of the flavonoids in the plant is similar in two different phenological stages, that is flowering and vegetative state [18]. 

Taking into account the abundance of flavonoids in the extracts prepared from *Ageratina havanensis,* it is expected that these extracts have antioxidant properties [19,20,21]. As qualitative composition of flavonoids is similar in both phenological stages, it could be hypothesized that the biological activity in both stages is similar, too. Based on these considerations, in this paper we studied the *Ageratina havanensis* (Kunth) R. M. King & H. Robinson extracts to prove their ability to inhibit P-gp function under no cytotoxicity conditions, their antioxidant potential and the influence of their quantitative composition on the biological properties of the plant. 

## 2. Results

### 2.1. P-gp Modulation by Extracts Obtained from Ageratina havanensis

The first step of this study was to investigate if the extracts obtained from *Ageratina havanensis* could inhibit P-gp activity under no cytotoxicity conditions. In order to mimic the chemo-resistance in humans, the cells chosen for this research were the well-characterized mouse mammary carcinoma 4T1 cells that express multi-resistance phenotype after exposure to different anticancer drugs mediated by P-gp. 

Firstly, to determine the cytotoxic effects of the eleven extracts obtained from *Ageratina havanensis* on 4T1 cells, the MTT assay was employed. Table 1 reports the IC_50_ values calculated after exposure of the cells to the *Ageratina havanensis* extracts for 24 h. As shown, the treatments reduced cell viability showing only slight differences between the products. In all the cases, significant differences were observed in comparison with control cells for values above 250 μg/mL. Thus, a range of concentrations under IC_50_ values was selected for evaluating effects of the extracts on P-gp function.

Rho-123 is a fluorescent compound, which enters the cells passively and concentrates in mitochondria. As P-gp substrate, the intracellular loading of this probe is inversely proportional to P-gp activity. Intracellular fluorescence increased in a dose-dependent fashion in 4T1 cells exposed to Lf-EtOH until F-EtOH extract for 1 h, but important differences between the extracts were found. The percentage of the inhibitory effect on the activity of the transporter produced at the highest concentration tested (200 μg/mL) are showed in Table 1. As reported, all ethanolic extracts (Lf-EtOH, Lv-EtOH, Sf-EtOH and F-EtOH) showed promissory inhibitory effects. More in details, the inhibitory activity was above 50% for both Sf-EtOH and Sv-EtOH, the two extracts obtained from the stem bark, compared to controls. On the contrary, the F-EtOAc and F-*n*-BuOH extracts were not able to inhibit the function of the transporter under the same experimental conditions.

### 2.2. Antioxidant Effects of Extracts Obtained from Ageratina havanensis

The screening of the antioxidant activity of the substances may require a combination of different methods to describe the background about the antioxidant properties of the samples. Here, the antioxidant potential of the *Ageratina havanensis* extracts was determined by using three in vitro methods, which previously have been used to predict the antioxidant capacity of several substances (DPPH free radical scavenging assay, FRAP assay and the determination of lipid peroxidation in brain rat homogenates) [22,23,24].

The model of scavenging the stable DPPH radical has been used method to evaluate the free radical scavenging ability of substances [23,24]. In this case, the antioxidant effect of the analyzed sample on DPPH radical scavenging may be due to their hydrogen donating ability and it reduce the stable violet DPPH radical to the yellow DPPH-H. Substances which are able to perform this reaction can be considered as antioxidants and therefore radical scavengers [25]. On the other hands, FRAP assay is based on the ability of antioxidant to reduce Fe^3+^ to Fe^2+^ in the presence of tripyridyltriazine (TPTZ), forming the intense blue Fe^2+^–TPTZ complex with an absorption maximum at 593 nm; the absorbance increase is proportional to the antioxidant content [22]. As shown in Table 2, the radical scavenging activity of the eleven *Ageratina havanensis* extracts evaluated was significantly (*p* < 0.05) higher in the flowering compared to the vegetative season, meanwhile, the reductive capacity was significantly (*p* < 0.05) higher in vegetative state. 

During the last years, lipid peroxidation has received renewed attention from the viewpoints of nutrition and medicine. Lipid peroxidation is implicated in the underlying mechanisms of several disorders and diseases such as cardiovascular diseases, cancer, neurodegenerative diseases, and even aging [26]. It is the accumulated result of reactive oxygen species and a chain reaction that causes the dysfunction of biological systems [27]. Furthermore, the extracts from the flowering season showed a significant (*p* < 0.05) inhibition of lipid peroxidation against brain phospholipid peroxidation compared with the extracts from the vegetative stage (Table 2).

### 2.3. Quantification of Sakuranetin and 7-Methoxyromadendrin in the Extracts of Ageratina havanensis

**Figure 1:**
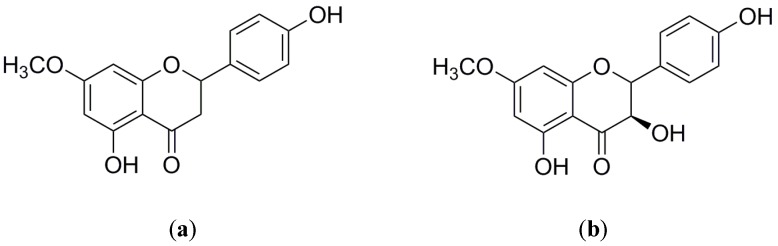
Chemical structures of: (**a**) sakuranetin; (**b**) 7-methoxyromadendrin.

The major flavonoids sakuranetin and 7-methoxyaromadendrin only differ for a hydroxyl group (Figure 1) and thus elute with very close retention times. Because of this, it was necessary to develop a rapid, sensitive and accurate method that would allow for their quantification in *Ageratina havanensis* extracts. UPLC-ESI-MS/MS system provides high separation capacity, high analytical speed and high analytical sensitivity. The addition of 0.1% formic acid to the mobile phase helped achieve satisfactory peak symmetry, good resolution, and significantly enhanced sensitivity. In this case, this method was useful for quantifying sakuranetin and 7-methoxyaromadendrin in the extracts of *Ageratina havanensis* collected in both seasons.

#### 2.3.1. Method Validation

##### Linearity, LDQ and LOQ

Calibration curves of the standard sakuranetin and 7-methoxyaromadendrin were established with negative UPLC-ESI-MS/MS on SRM mode. The validation data of the method showed good correlation coefficients, 0.9909 and 0.9928, respectively, in the mass concentration range of 1–50 ppm, Table S1. 

##### Matrix Effect and Recovery

The recoveries after SPE were 93.2% and 97.4% for sakuranetin and 7-methoxyaromadendrin respectively, which means that 6.8 and 2.6% were lost in the solid phase extraction process. The ratios (A/B × 100)% were 88.3% for sakuranetin and 94.7% for 7-methoxyaromadendrin, so the presence of the matrix decreases the recovery of sakuranetin and 7-methoxyaromadendrin by 4.9% and 2.7% respectively. These results showed that all values were within acceptable ranges.

##### Precision and Accuracy of the System

In this method, injection errors, dilution, adduct formation etc. can occur. Therefore, precision and accuracy of the system were evaluated. The results suggest that both parameters were acceptable for the quantification method developed. In the case of precision, very similar % RSD were observed. The results of sakuranetin were less than 2.5% in the experiments intra-day and inter-day whereas for 7-methoxyaromadendrine were less than 3.5%. Accuracy showed results above 98% intra-day and inter-day in both standards for the concentrations tested, Table S2.

#### 2.3.2. Concentration of Standards in the Extracts

The linear calibration curves were used for quantitative analyses of the standards in the extracts, Table 3. In general, the ethyl acetate extracts showed higher concentration of the analyzed patterns. The concentration of sakuranetin is approximately three times the concentration of 7-methoxyarycomadendrine. Extracts from the leaves of the vegetative season present a higher concentration of analyzed flavonoids than those from the flowering season.

#### 2.3.3. Principal Component Analysis of Extracts

Principal component analysis (PCA) allowed comparing extracts of *Ageratina havanensis* according their chemical composition. The original variables were reduced to the two principal components PC1 (77.9%) and PC2 (5.6%) representing an 83.6% of the total data variance (Table S3). According to eigenvalues, peaks with *m*/*z* 447 gave principal contribution to PC1. Also, sakuranetin (*m*/*z* 285, [M − H]^−^), 7-methoxyaromadendrin (*m*/*z* 301, [M − H]^−^) and peaks with *m*/*z* 463 had great influence on the variability of the data in this component. From the extracts of *Ageratina havanensis,* were isolated and identified in previous studies three glycosides with *m*/*z* 447 and the same fragmentation ([286 + 162 − H]^−^) in its MS^2^ spectrum [17,18]. Because of that, it was concluded that the most influential variables in PC1 were the concentration of sakuranetin and 7-methoxy-aromadendrin. On the other hand, the glycosides (*m*/*z* 447 and *m*/*z* 463) were the variables that most influenced on PC2, Table S4.

**Figure 2:**
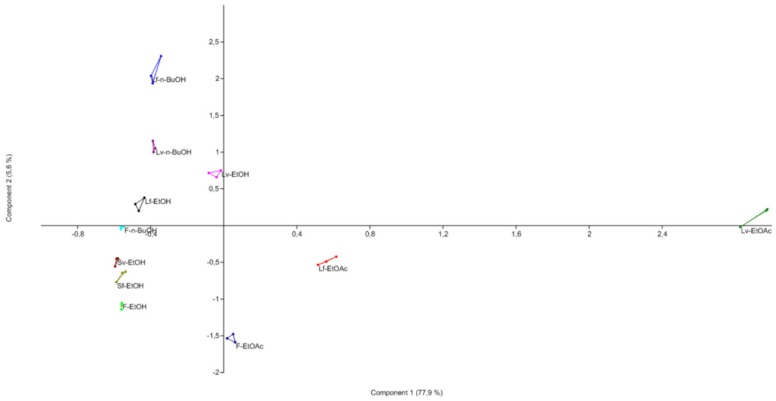
Scatter plot of PCA of the extracts on the first two principle components.

Figure 2 shows that the ethyl acetate extract from the leaves collected in vegetative state (Lv-EtOAc) differs from the rest of the extracts with respect to PC1 followed by the ethyl acetate extract from the leaves collected in flowering state (Lf-EtOAc). The rest of the extracts showed similarity in PC1. According to this behavior, it was concluded that sakuranetin, 7-methoxyaromadendrin and the glycosides (the original variables that most influence CP1) were higher in the vegetative stage, mainly in the ethyl acetate extract of leaves. With respect to PC2, the *n*-butanol extract of the leaves collected in flowering state (Lf-*n*-BuOH) is different from the rest of the extracts. Sakuranetin and 7-methoxyaromadendrin in this case do not influence the total variability of the data but the glycosides have a significant influence. These results were expected since in the quantification, the concentration of both sakuranetin and 7-methoxyaromadendrin resulted higher in the extracts of ethyl acetate of the leaves, being greater in the vegetative state, whereas in the ethanolic and *n*-butanolic extracts, these flavonoids were below the limit of quantification.

## 3. Discussion

Breast cancer is the most frequently diagnosed cancer and the leading cause of cancer-related death among women worldwide [28]. Despite the long list of drugs that have been used in the treatment of breast tumors, marked drug multi-resistance during treatment remains one of the main problems facing the clinic today when applying a therapy against breast cancer. Among various mechanisms of chemo-resistance, resistance due to the increased expression of P-gp is the best characterized and it is considered the most important in cancer therapy. The significance of the above mentioned is also supported by the fact that more than 50% of existing anticancer drugs are going to P-gp mediated efflux [29]. Since the results of clinical trials using P-gp inhibitors have not been very promising, the search for new modulators which selectively inhibit P-gp activity without significant negative side effects is currently under high interest and hopes are pinned on the application of some plant polyphenols [11]. 

P-gp may affect the pharmacokinetic parameters of drugs or other compounds, possibly leading to modifications of their bioavailability as well as of their distribution, metabolism, elimination and toxicity (ADMET) [30]. Together with several CYP450 isoenzymes, P-gp is involved in drug-drug, food-drug and, finally, herb-drug interactions [31]. Herbal medicines may interact with drugs at the intestine, liver, kidneys, and targets of action. Importantly, many of these drugs have very narrow therapeutic indices. Most of them are substrates for P-gp. The underlying mechanisms for most described herb-drug interactions are not entirely understood, and several pharmacokinetic and/or pharmacodynamic interferences are often implicated in these interactions. In particular, enzyme induction and inhibition may play an important role in the occurrence of some herb-drug interactions. Because herb-drug interactions can significantly affect circulating levels of drug and, hence, alter the clinical outcome, the identification of herb-drug interactions has important implications [32]. As present results suggest the species *Ageratina havanensis* (Kunth) R. M. King & H. Robinson could be a source of new potential inhibitors of drug efflux mediated by P-gp; focus on these aspects must be taking into account for future studies.

Today it is known that P-gp function and its regulation can be mediated via reactive oxidative species (ROS) [33,34,35,36,37]. Therefore, the transport of P-gp substrates may be altered by modulation of P-gp expression/activity under conditions of oxidative stress [38]. In this sense, natural antioxidants, such as polyphenols, could influence the function of this membrane transporter. It was reported that polyphenolic compounds, mainly flavonoids or their derivatives, can modulate the main ABC transporters responsible for cancer drug resistance, including P-gp [16,39,40,41]. 

The interest in health benefits of polyphenols has increased due to their powerful antioxidant and free radical scavenging activities observed in vitro [42]. Current evidence strongly supports a contribution of polyphenols, in particular flavonoids, to the prevention and therapy of cancer and other chronic diseases [1,43]. Literature data have shown that plant phenolic content and antioxidant activity depend on several factors, mainly environmental conditions [3]. Previous studies show that summer plants (reproductive stage) are richer in phenolic compounds than spring ones (vegetative stage), and consequently exhibit higher antioxidant activities [2,3]. Here, we evaluated the antioxidant potential of the *Ageratina havanensis* extracts by using three in vitro methods of well-established use to strongly predict the antioxidant capacity of several substances [22,23,24]. But surprisingly, the results of the performed studies showed the opposite, the concentration of flavonoids is higher in the vegetative state. In fact, we observed that *Ageratina havanensis* extracts possess higher reductive capacity in the vegetative period. This finding might be related to the content of sakuranetin and 7-methoxyaromadendrin, which is greater in the vegetative stage. The antioxidant capacity of sakuranetin has been previously demonstrated [44]. Indeed, here we observed that sakuranetin possesses the greatest reductive capacity of the analyzed samples. In order to evaluate the antioxidant capacity in a biological system, we used the lipid peroxidation model in brain homogenates. Rat brain homogenates exposed to oxygen spontaneously exhibit lipid peroxidation by a mechanism which is independent of superoxide and free hydroxyl radical production and whose initiation step may involve and iron-mediated cleavage of lipid hydroperoxides to yield peroxide or alkoxy radicals [45,46]. In this system, the extracts obtained from the flowering stage effectively inhibited TBARS generation with estimated values (see Table 1). This result could be explained in term of several mechanisms comprising among others, iron chelation, (at the initiation step) or a synergistic antioxidant activity (at the propagation step). In addition, previous studies demonstrated the metal chelation properties of polyphenols [47,48]. The present results suggest that *Ageratina havanensis* could be represent a source of antioxidants with potential neuroprotective actions. In summary, these results show for first time the antioxidant potential of the extracts obtained from the species *Ageratina havanensis*.

## 4. Materials and Methods 

### 4.1. General

Analytical-grade *n*-hexane, ethyl acetate (EtOAc), *n*-butanol (*n*-BuOH), ethanol (EtOH) and UPLC/MS-grade methanol (Sigma Aldrich, St. Luis, MO, USA) were used. HPLC-grade water was prepared using a Milli-Q purification system (Millipore, Burlington, MA, USA). The RP18 cartridge was a Phenomenex Strata C18-E. Sakuranetin and 7-methoxyaromadendrin (standards) were previously isolated from the leaves of *Ageratina havanensis* (Kunth) R. M. King & H. Robinson [17]. They were identified and standardized by spectroscopic techniques (^1^H-NMR, ^13^C-NMR, 2D NMR, MS). The stability was evaluated by NMR.

### 4.2. Plant Material and Extraction

*Ageratina havanensis* (Kunth) R. M. King & H. Robinson (Asteraceae) as authenticated by Prof. Iralys Ventosa, Instituto de Ecología y Sistemática, Havana, Cuba (voucher herbarium specimen number HAC-42498) was collected in Havana (East Havana, Alamar neighbourhood) during 2012 in flowering and vegetative stage. The secondary metabolites were extracted as previously reported [17,18].

### 4.3. Cytotoxicity and P-glycoprotein Activity Assays 

The study was conducted using 4T1 cells (ATCC, Manasssas, VA, USA) cultured in RPMI-1640 medium containing 10% fetal bovine serum, 1% penicillin-streptomycin and glutamine (2 mmol/L) at 37 °C in 5% CO_2_ humidified incubator. 

Cytotoxicity was measured using the MTT reduction assay [49]. After extracts exposure, cells were washed and a 100 µL/well of MTT reagent (5 mg/mL) was added. After a 4 h incubation period at 37 °C, supernatants were discarded. The dye was extracted with dimethyl sulfoxide and optical density was read at 540 nm. The inhibition of reduction of MTT was expressed as the percentage of viable cells referred to control cells, those incubated in presence of the vehicle only (100% viability value). IC_50_ values were calculated.

P-glycoprotein (P-gp) activity was evaluated by the Rhodamine-123 (Rho-123) accumulation assay [13]. Cells (4 × 10^4^/well) were treated for 24 h with sub-toxic concentrations of the extracts (10–200 µg/mL) or verapamil (20 µM), an inhibitor of P-gp activity (positive control). Cells were washed and incubated with 5 µg/mL Rho-123 for 2 h. Afterwards, the cells were lysed with 0.1% Triton X-100. In cell lysates fluorescence intensity was measured at the 485 nm excitation and 535 nm emission wavelengths. Data were expressed as percentage of fluorescence relative to control cells.

### 4.4. Antioxidant Capacity Assays

The free radicals scavenging capacity of the extracts was determined [50]. For it, an ethanolic solution of 130 mM 2,2-diphenyl-1-picrylhydrazyl (DPPH^•^, Sigma Aldrich, St. Luis, MO, USA) was mixed with the extracts (2–1000 µg/mL). Ascorbic acid (Sigma) 50 µg/mL was employed as standard. The reaction mixtures were incubated in the dark at room temperature for 30 min and the absorbance was measured at 515 nm. The inhibition percent of DPPH^•^ radical was calculated by:

Inhibition (%) = (D.O. control − D.O. sample)/D.O. control) × 100(1)

The concentration required to scavenge 50% of DPPH (IC50) was determined.

The reducing capacity of the plant extracts was measured according to the method of Benzie and Strain (1996) [51]. Briefly, acetate buffer (300 mM, pH = 3.6), TPTZ (2, 4, 6-tripyridyl-s-triazine; Sigma) 10 mM in 40 mM HCl and FeCl_3_ × 6H_2_O (20 mM) were mixed in the ratio of 10:1:1 to obtain FRAP reagent. The extracts (20 µL) were mixed with 900 µL of FRAP reagent. The mixtures were incubated at room temperature for 4 min and absorbances were measured at 593 nm. Ascorbic acid (100 µM) was used as standard.

To evaluate the inhibition of brain phospholipid peroxidation, rat brains were excised after decapitation, weighed and washed with 0.9% NaCl ice cold solution. Tissue homogenates were prepared in a ratio of 1 g of wet tissue to 9 mL of phosphate buffer (50 mM, pH 7.4), by using a tissue homogenizer (Qiagen). The homogenates were centrifuged at 800× *g* in a Sigma centrifuge at −4 °C during 15 min and the supernatants were kept at −70 °C until analysis. Thiobarbituric reactive substances (TBARS) measurement assay was carried out as previously described [52]. Brain homogenates (25 mL) were incubated with different extracts (5–125 µg/mL). Incubations were stopped by the addition of 350 mL of cold acetic acid 20% pH 3.5. Malondialdehyde (MDA) levels were determined by the addition of 600 mL of TBA 0.5% in acetic acid 20% pH 3.5. The mixtures were incubated at 90 °C for 1 h. Then, 50 mL of sodium dodecyl sulfate (SDS) were added, and samples were centrifuged at 500× *g* during 15 min at room temperature. The absorbance was measured at 532 nm. All the values are means of three determinations. Trolox-C (0.5–75 µg/mL), was used as standard. The extract concentration which is needed to achieve the 50% of inhibition of lipid peroxidation (IC50) was calculated.

### 4.5. Statistical Analysis

Values were expressed as mean ± standard error of mean (SEM). Statistical analysis was performed with SPSS 18.0 (SPSS Inc., Chicago, IL, USA). For multiple comparisons, one-way ANOVA was used followed by Dunnett post-hoc test. Values of *p* < 0.05 were considered statistically significant.

### 4.6. Sample Preparation for UPLC-ESI-MS/MS Analysis

A solution of each extract (MeOH/H_2_O 8:2 *v*/*v*, 1 mg/mL) was submitted to the solid-phase extraction using RP18 cartridge, eluted with MeOH/H_2_O 8:2 (*v*/*v*). After drying, 1 mg was dissolved in 1 mL of MeOH/H_2_O 8:2 (*v*/*v*) solution (solution A) and an aliquot was diluted with MeOH/H_2_O 8:2 (*v*/*v*) to reach a final volume of 1 mL (150 μg/mL), and was filtered through a 0.22 μm nylon filter membrane. The final solution was used for quantification analysis. For PCA, the solution A was diluted with MeOH/H_2_O 8:2 (*v*/*v*) to reach a final concentration of 1 μg/mL. The sample preparations were triplicated.

#### Standard Solutions Preparation for Quantification Analysis

Sakuranetin and 7-methoxyaromadendrin were dissolved in 1 mL of MeOH/H_2_O 8:2 (*v*/*v*) solution (1 mg/mL) and filtered through a 0.22 μm nylon filter membrane. An aliquot was diluted with MeOH/H_2_O 8:2 (*v*/*v*) up to a volume of 1 mL (100 ppm, solution A). The solution A was diluted to obtain the solutions patterns between 1 and 50 μg/mL (three replicas for each concentration).

### 4.7. UPLC-ESI-MS Analysis of the Extracts

Analysis was performed on a Waters^®^ Acquity UPLC system coupled with a XevoTqD^®^ mass spectrometer (Waters Corp., Milford, MA, USA). The analytical column used was an ACQUITY^TM^ UPLC X bridge C18 (2.1 × 50 mm, 2.5 μm). Analysis was carried out with water containing 0.1% formic acid (A) and methanol (B) at a flow rate of 400 μL/min during 10 min. A gradient program was used for quantification as follows: 0–8 min, 47% B isocratic; 8–10 min, 47–100% B linear and for PCA: 0–3.2 min, 40% B isocratic; 3.2–4 min, 40–60% B linear; 4–8.2 min, 60% B isocratic; 8.2–10 min, 60–100% B linear. The injection volume was 5 μL in quantification analysis and 10 μL in PCA analysis. Mass spectrometric detection was in negative mode. The MS conditions were as follows: capillary temperature 200 °C; capillary voltage 3.5 kV; Cone voltage 39 kV; desolvation temperature 200 °C; Source gas flow: desolvation 400 L/h. Selected reaction monitoring mode was used for quantification. The monitored transitions included the following: sakuranetin (285 > 165) and 7-methoxyaromadendrin (301 > 165). All data were acquired and processed using the Masslynx^TM^ V4.1 software (Waters Corporation, Milford, MA, USA).

#### 4.7.1. Quantification Method Validation

##### Linearity, LDQ and LOQ

Calibration curves were generated by using 1/x-weighted least-square linear regression. Concentrations of the flavonoids were calculated by linear interpolation from the calibration curves. LDQ was determined by the following calculation: (Y_bl_ + 3S_bl_)/b, while LOQ was calculated multiplying Sbl by 10 [53].

##### Matrix Effect and Recovery

Matrix effects were determined by comparing the peak area of standards in the extracts (A), after evaluation of a sample target, with that of analyte standard solutions (B). The recoveries of the analytes were determined by comparing the peak area of analytes standard solution (8 ppm) after solid phase extraction (SPE) with that of the analytes’ standard solution (8 ppm) before SPE. The ratio (after SPE/before SPE) × 100 was determined.

##### Precision and Accuracy of the System

Precision and accuracy were assessed on three consecutive days by using mixtures containing low, medium, or high concentrations of the analytes’ standard solution. Precision was expressed as the RSD %, and accuracy (% RE) was calculated using the following equation:

[(mean measured concentration−concentration of standard solutions)/(concentration of standard solutions)] × 100.(2)

#### 4.7.2. PCA of Extracts

The software Markerlynx^XS^ (Waters Corporation, Milford, MA, USA) was used to generate a matrix containing the *m/z* ratio of ions detected in each sample and the area under the curve of each peak in the chromatogram. PCA calculation was performed using Past 2.14 software (Waters Corporation). PC to be analyzed were selected according to the significance of the eigenvalues of each component. The loads were considered to evaluate the impact of the original variables on the new components, while percent of variance explained of the new PC selected were used to evaluate similarities and differences between the extracts studied.

## 5. Conclusions

In this work, a rapid and sensitive method was developed to quantify the major flavonoids present in eleven *Ageratina havanensis* extracts obtained from two phenological states of *Ageratina havanensis*. The phenological state of the plant influences the concentration of sakuranetin, 7-methoxyaromadendrine and glycosides with *m*/*z* 447 in its extracts; in fact, the content of these compounds is higher in the vegetative state. In addition, the antioxidant potential of *Ageratina havanensis* (Kunth) R. M. King & H. Robinson extracts and its relationship with the concentration of major flavonoids were demonstrated for the first time. The results obtained in this study also highlight that this plant could be a source of new potential inhibitors of drug efflux mediated by P-gp. However, other in vitro and in vivo studies must be conducted to confirm the potential pharmacological or therapeutic relevance of these findings. 

## Figures and Tables

**Table 1:** Cytotoxicity and inhibitory effects on P-gp function of *Ageratina havanensis* (Kunth) R. M. King & H. Robinson extracts on breast cancer 4T1 cells.

Harvesting Stage	Plant Organs	Extracts	IC_50_ (μg/mL)	Inhibition P-gp Function (%)
Flowering	Leaves	Lf-EtOH	381.6 ± 7.5	35
		Lf-EtOAc	252.5 ± 10.1	23
		Lf-*n*-BuOH	302.0 ± 8.0	19
Vegetative stage		Lv-EtOH	392.8 ± 6.7	58
		Lv-EtOAc	313.0 ± 12.1	45
		Lv-*n*-BuOH	496.5 ± 6.7	27
Flowering	Stems	Sf-EtOH	228.2 ± 8.7	84
Vegetative stage		Sv-EtOH	355.7 ± 7.6	65
Flowering	Flowers	F-EtOH	263.5 ± 8.2	85
		F-EtOAc	259.5 ± 10.6	-
		F-*n*-BuOH	315.5 ± 9.9	-

IC_50_ is defined, as the concentration required achieving 50% inhibition over control cells, values are shown as mean ± SEM. %: represents the percentage of inhibition P-gp activity at the higher concentration tested (200 μg/mL) respect to control cells (untreated cells) after 1 h of exposure. Verapamil used as control positive was included, showing values of inhibition in the order of 300% to the activity exhibit by control cells. Values showed are from two independent experiments with three replicas.

**Table 2:** In vitro antioxidant capacity of *Ageratina havanensis* (Kunth) R. M. King & H. Robinson extracts.

Harvesting Stage	Plant Organs	Extracts	FRAP (μM of Ascorbic Acid Equivalents)	DPPH IC_50_ (μg/mL)	Lipid Peroxidation Inhibition IC_50_ (µg/mL)
Flowering	Leaves	Lf-EtOH	88.21 ± 3.77 ^a^	22.65 ± 1.73 ^a^	16.52 ± 2.19 ^a^
		Lf-EtOAc	90.52 ± 3.61 ^a^	39.27 ± 2.04 ^a^	21.63 ± 1.78 ^a^
		Lf-*n*-BuOH	63.51 ± 2.62 ^a^	53.12 ± 0.92 ^a^	38.91 ± 3.52 ^a^
Vegetative stage		Lv-EtOH	279.42 ± 8.48 ^b^	151.90 ± 3.71 ^b^	41.66 ± 2.87 ^b^
		Lv-EtOAc	234.70 ± 7.93 ^b^	138.11 ± 2.67 ^b^	46.23 ± 3.12 ^b^
		Lv-*n*-BuOH	231.56 ± 10.14 ^b^	84.24 ± 3.81 ^b^	51.60 ± 2.04 ^b^
Flowering	Stems	Sf-EtOH	88.43 ± 3.96 ^a^	39.23 ± 1.92 ^a^	37.21 ± 2.55 ^a^
Vegetative stage		Sv-EtOH	197.21 ± 3.30 ^b^	32.72 ± 7.23 ^a^	48.32 ± 3.87 ^b^
Flowering	Flowers	F-EtOH	169.91 ± 7.63 ^a^	29.45 ± 1.99 ^a^	21.35 ± 2.66 ^a^
		F-EtOAc	167.21 ± 5.34 ^a^	38.77 ± 2.64 ^b^	32.34 ± 2.01 ^b^
		F-*n*-BuOH	91.66 ± 3.21 ^b^	54.13 ± 2.88 ^c^	30.27 ± 3.81 ^b^
Sakuranetin			319.38 ± 6.65	-	-
7-methoxyaromadendrin			238.45 ± 2.11	-	-
Ascorbic acid			-	21.97 ± 0.84	-
Trolox-C					10.64 ± 1.02

Values represent the mean ± SEM of the antioxidant activity of *A. havanensis* extracts. IC_50_ values were calculated as the extract concentration required to scavenge 50% of DPPH^•^, Ascorbic acid was employed as standard for DPPH^•^ assay and Trolox-C for lipid peroxidation assay. Different letters in the same column represent statistical differences (ANOVA, Dunnet post-hoc test; *p* < 0.05). Comparisons were carried out between extracts according to the seasonal stage (leaves, stems or flowers) or according to the solvent (EtOH, EtOAc or *n*-BuOH). Three independent experiments were done and samples were analyzed by triplicate.

**Table 3:** Concentration of sakuranetin and 7-methoxyaromadendrin in the extracts by UPLC/ESI/TQD/MS^n^.

Harvesting Stage	Plant Organs	Extracts	Concentration (μg/mL)	
			Sakuranetin	7-Methoxyaromadendrin
Flowering	Leaves	Lf-EtOH	Below LOQ	1.27
		Lf-EtOAc	46.70	16.8
		Lf-*n*-BuOH	Below LOQ	Below LOQ
Vegetative stage		Lv-EtOH	13.20	2.90
		Lv-EtOAc	87.02	30.2
		Lv-*n*-BuOH	Below LOQ	Below LOQ
Flowering	Stems	Sf-EtOH	Below LOQ	Below LOQ
Vegetative stage		Sv-EtOH	Below LOQ	Below LOQ
Flowering	Flowers	F-EtOH	Below LOQ	Below LOQ
		F-EtOAc	29.90	6.30
		F-*n*-BuOH	Below LOQ	Below LOQ

## Supplementary Materials

The following are available online. Table S1: Equations for calibration curves, limit of detection (LDQ) and limit of quantitation (LOQ) by UPLC/ESI/TQD/MS^n^. Table S2: Summary of precision and accuracy results by UPLC/ESI/TQD/MS^n^, Table S3: Principal components selected for the PCA, Table S4: Most influential variables in PC. Figure S1: Chromatogram of sakuranetin at 25 μg/mL, Figure S2: Chromatogram of 7-methoxyaromadendrin at 25 μg/mL, Figure S3: MRM chromatogram of 7-methoxyaromadendrin and sakuranetin in the ethyl acetate extract of *Ageratina havanensis* leaves.
