# Supplementary material for: Influence of the Phenological State of in the Antioxidant Potential and Chemical Composition of Ageratina havanensis. Effects on the P-Glycoprotein Function
Source: Molecules. 2020 May 2;25(9):2134. doi: 10.3390/molecules25092134 (PMC7248889; doi:10.3390/molecules25092134)
Supplement: Supplementary file 1 [file molecules-25-02134-s001.pdf]

Supplementary material

Influence of the phenological state of *Ageratina havanensis* on the quantitative chemical composition and antioxidant potential. Effects on the P-glycoprotein function

Table S1. Equations for calibration curves, limit of detection (LOD) and limit of quantitation (LOQ) by UPLC/ESI/TQD/MS^n

Standard	Calibration curves	Linear range (ug/mL)	r^2	LDQ (ug/mL)	LOQ (ug/mL)
sakuranetin	y= 3773.5x + 10742	1-50	0.9909	2.85	2.86
7-methoxyaromadendrin	y= 899.67x + 2154.8	1-50	0.9928	2.60	3.08

Table S2. Summary of precision and accuracy results by UPLC/ESI/TQD/MS^n

Standards	Concentration (μg/mL)	Intra-day precision (%, RSD)	Intra-day accuracy (%, RE)	Inter-day precision (%, RSD)	Inter-day accuracy (%, RE)
sakuranetin	1	1.7	101.1	2.0	98.7
	12	1.9	99.7	2.4	98.0
	50	2.1	99.9	1.9	99.2
7-methoxy aromadendrin	1	3.1	95.5	2.6	94.5
	12	2.9	95.9	3.1	92.9
	50	2.7	94.7	2.7	94.2

Table S3. Principal components selected for the PCA

PC	Eigenvalue	% variance
1	1.52226E09	77.9
2	1.1005E08	5.6

Table S4. Most influential variables in PC

PC	Original variables	
	Variable $[M-H]^-$	Eigenvector
PC1	447	0.7043
	285	0.4330
	301	0.3702
	463	0.1334
PC2	463	0.3670
	447	0.1429

Figure S1. Chromatogram of sakuranetin at 25 μg/mL

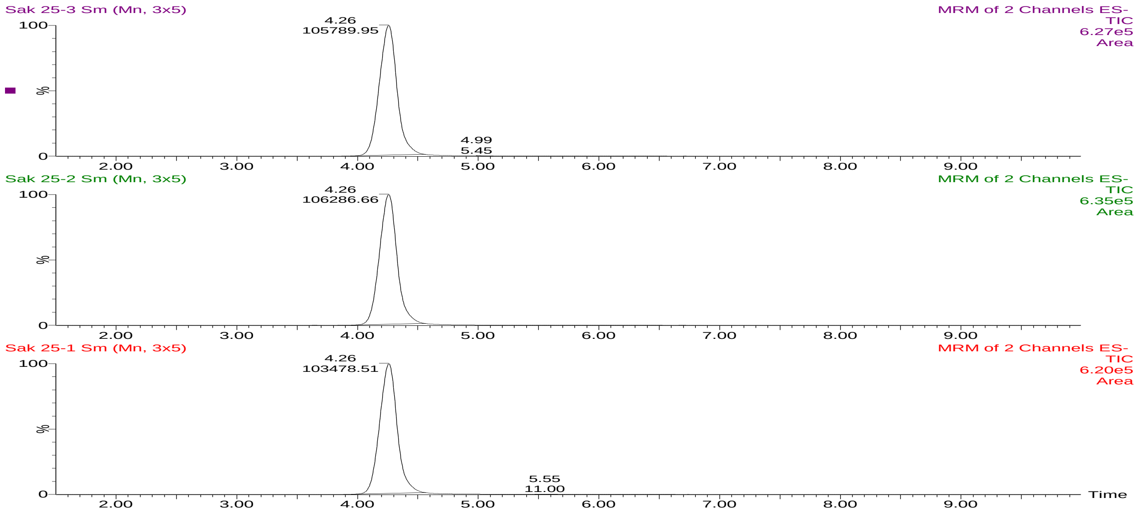

Figure S2. Chromatogram of 7-methoxyaromadendrin at 25 μg/mL

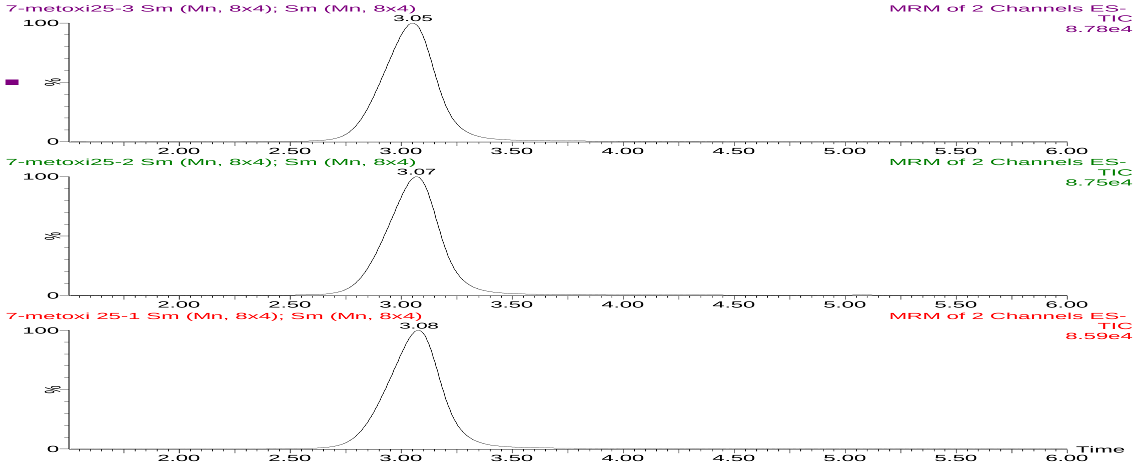

The figures S1 and S2 show the chromatograms of the pure compounds used in the quantification curve.

Figure S3. MRM chromatogram of 7-methoxyaromadendrin and sakuranetin in the ethyl acetate extract of *Ageratina havanensis* leaves

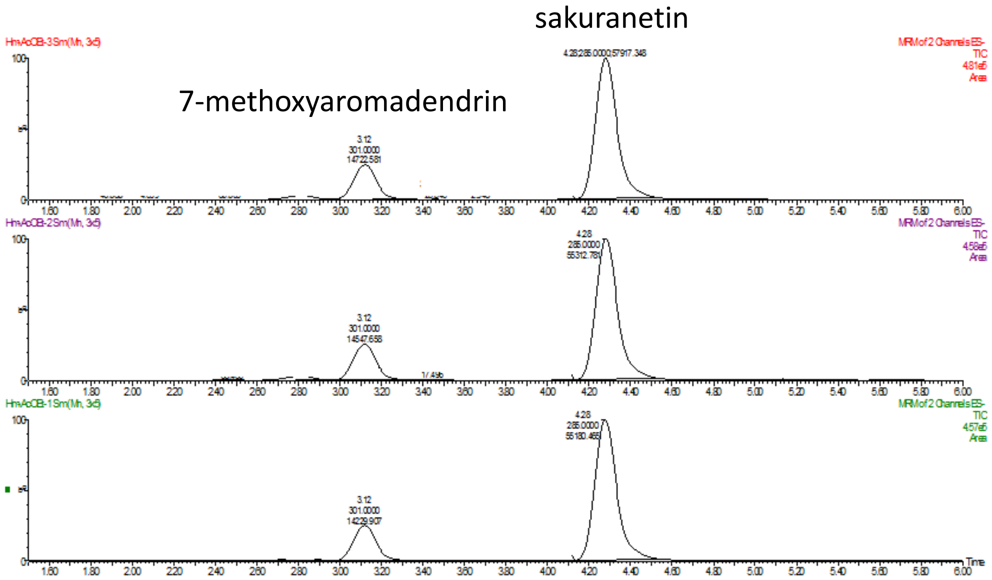

The figure **S3** shows the MRM transition of the compounds in the Ethyl Acetate extract, proving that there are compounds 7-methoxyaromadendrin and sakuranetin in extract.
